# Reef Endemism, Host Specificity and Temporal Stability in Populations of Symbiotic Dinoflagellates from Two Ecologically Dominant Caribbean Corals

**DOI:** 10.1371/journal.pone.0006262

**Published:** 2009-07-15

**Authors:** Daniel J. Thornhill, Yu Xiang, William K. Fitt, Scott R. Santos

**Affiliations:** 1 Department of Biological Sciences, Auburn University, Auburn, Alabama, United States of America; 2 Department of Biology, Bowdoin College, Brunswick, Maine, United States of America; 3 Odum School of Ecology, University of Georgia, Athens, Georgia, United States of America; Northeastern University, United States of America

## Abstract

**Background:**

The dinoflagellate genus *Symbiodinium* forms symbioses with numerous protistan and invertebrate metazoan hosts. However, few data on symbiont genetic structure are available, hindering predictions of how these populations and their host associations will fair in the face of global climate change.

**Methodology/Principal Findings:**

Here, *Symbiodinium* population structure from two of the Caribbean's ecologically dominant scleractinian corals, *Montastraea faveolata* and *M. annularis*, was examined. Tagged colonies on Florida Keys and Bahamian (i.e., Exuma Cays) reefs were sampled from 2003–2005 and their *Symbiodinium* diversity assessed via internal transcribed spacer 2 (ITS2) rDNA and three *Symbiodinium* Clade B-specific microsatellite loci. Generally, the majority of host individuals at a site harbored an identical *Symbiodinium* ITS2 “type” B1 microsatellite genotype. Notably, symbiont genotypes were largely reef endemic, suggesting a near absence of dispersal between populations. Relative to the Bahamas, sympatric *M. faveolata* and *M. annularis* in the Florida Keys harbored unique *Symbiodinium* populations, implying regional host specificity in these relationships. Furthermore, within-colony *Symbiodinium* population structure remained stable through time and environmental perturbation, including a prolonged bleaching event in 2005.

**Conclusions/Significance:**

Taken together, the population-level endemism, specificity and stability exhibited by *Symbiodinium* raises concerns about the long-term adaptive capacity and persistence of these symbioses in an uncertain future of climate change.

## Introduction

Determining the population dynamics of marine organisms is critical toward understanding their evolutionary ecology as well as successfully designing marine protected areas (MPAs) [1]–[2]. For coral reef ecosystems, genetic studies have revealed the degree of gene flow and connectivity in many species, such as fish [e.g., 3]–[5] and numerous other reef dwelling metazoans [e.g., 6]–[7], including scleractinian corals [e.g., 8]–[10]. However, surprisingly little is known about the population genetics of *Symbiodinium*, the unicellular symbiotic dinoflagellates that are among coral reefs' most important constituents [11]. This is particularly relevant since rising sea-surface temperatures are increasingly threatening coral reef ecosystems and populations that reside within them [reviewed by 12]. Since populations represent the fundamental units of evolution [reviewed by 13], understanding patterns and processes at this level is paramount toward furthering our knowledge on the basic biology of *Symbiodinium* as well as how anthropogenically driven global climate change may impact these symbionts and their host associations in the future.

*Symbiodinium* forms symbioses with a variety of marine protistan and invertebrate metazoan hosts. Currently, the genus is viewed as ecologically, physiologically, biochemically, and genetically diverse [e.g., 14]–[20]. Although previous genetic studies have extensively examined symbiont diversity at the levels of sub-generic “Clades”, chloroplast and mitochondrial “haplotypes”, or internal transcribed spacer (ITS) rDNA “types” [reviewed by 21], only five studies to date have examined *Symbiodinium* at the population level. In this context, Santos et al. [22] reported that ITS2 “type” B1 [ITS2 “type” nomenclature *sensu* 18] *Symbiodinium* populations associated with the Caribbean gorgonian *Pseudopterogorgia elisabethae* exhibited strong genetic structure over distances of tens of kilometers in the Bahamas. Similarly, Kirk et al. [23]–[24] found significant differentiation between *Symbiodinium* “type” B1 populations of the sea fan *Gorgonia ventalina* on reefs separated by 5 to 200 km in the Florida Keys. In the Pacific Ocean, Howells et al. [25] reported that *Symbiodinium* Clade C populations associated with an alcyonacean coral, *Sinularia flexibilis*, were genetically differentiated at a range of spatial scales, from 16 to 1,360 km. On the other hand, Magalon et al. [26] observed no population structure in *Symbiodinium* Clade C from the scleractinian *Pocillopora meandrina* over distances of up to 200 km in the central and western Pacific Ocean. These disparate results may be a consequence of differences in host affiliation, *Symbiodinium* lineage, mode of symbiont acquisition, geographic/hydrological differences (such as currents), and/or other factors. Thus, additional studies are necessary to better elucidate patterns of *Symbiodinium* structure and gene flow at the population level. Here, we contribute to this area by examining the *Symbiodinium* Clade B populations associated with the scleractinian corals *Montastraea faveolata* (Ellis and Solander, 1786) and *Montastraea annularis* (Ellis and Solander, 1786).

*Montastraea faveolata* and *M. annularis* are two of the ecologically dominant reef-building corals in the Caribbean and western Atlantic Ocean, with both species acquiring their *Symbiodinium* from the external environment at each generation [27]. These coral species are among the most flexible hosts known; symbioses with various ITS2 “types” in *Symbiodinium* Clades A, B, C, and D [19], [28]–[32], along with intracolonial zonation of *Symbiodinium* Clades and “types” [28]–[29], [32], have been well-documented across their biogeographic range. Thus, these coral species provide an ideal system for studying population-level dynamics in *Symbiodinium*. Previously, Thornhill et al. [31], [33] tracked symbiotic associations in tagged colonies of nine coral species, including *M. faveolata* and *M. annularis*. At the ITS2-rDNA level, these hosts were found to engage in relatively stable temporal associations with particular *Symbiodinium*, such as “type” B1. This observation, along with earlier population genetic and host association studies of this symbiont “type” in the western Atlantic Ocean [22]–[24], [34], led us to hypothesize that the *Symbiodinium* “type” B1 populations of *M. faveolata* and *M. annularis* would exhibit endemism to particular reefs, host specificity and within-colony stability through time. To test these predications, microsatellite allelic variation at loci specific to *Symbiodinium* Clade B was 1) examined from symbiont populations of these scleractinian species on Florida Keys and Bahamian reefs, and; 2) tracked over an approximately three-year period.

## Methods

### Study sites and collection of *Montastraea faveolata* and *M. annularis*

**Figure 1:**
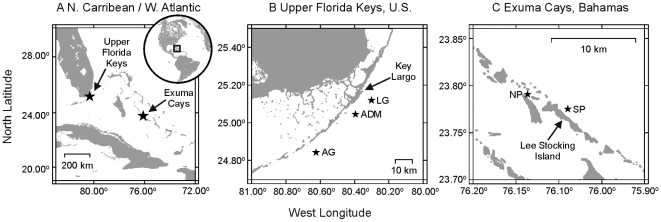
Maps of coral reefs sampled in this study. Regional (A) and local (B–C) maps of collection sites for *Montastraea faveolata* and *M. annularis*. Reef abbreviations are as follows: Upper Florida Keys (U.S.): LG = Little Grecian Reef, ADM = Admiral Patch Reef, AG = Alligator Reef; Exuma Cays (Bahamas): NP = North Norman's Patch Reef, SP = South Perry Reef.

Three reefs in the Upper Florida Keys, U.S. and two reefs in the Exuma Cays, Bahamas were selected for monitoring of symbiotic associations (Fig. 1). Those in the Florida Keys included the inshore Admiral Patch Reef (ADM; 1–2 m depth; 25.0446°N, 80.3945°W), offshore Little Grecian Reef (LG; 3–4 m depth; 25.1188°N, 80.3016°W), and the deeper-water Alligator Reef (AG; 12 m depth; 24.8424°N, 80.6244°W). Similarly, in the Exuma Cays, the inshore North Norman's Patch (NP; 2–4 m depth; 23.7912°N, 76.1368°W) and deeper-water South Perry Reef (SP; 12–15 m depth; 23.7752°N, 76.0895°W) were sampled. For further information on the history of coral colonies at these reefs, see Fitt et al. [35] and Thornhill et al. [31]. Nocturnal sea-surface temperature data for the study sites were obtained from the National Oceanographic and Atmospheric Administration's (NOAA) satellite-based Coral Reef Watch virtual and experimental coral bleaching stations (http://coralreefwatch.noaa.gov/satellite/index.html).

To ensure that subsequent sample collections were from the same individual, six colonies of *Montastraea faveolata* and *M. annularis*, each separated by 4 to 50 m from the nearest conspecific individual, were tagged on each reef (a total of 30 for *M. faveolata* and 30 for *M. annularis*). Note that in the genetic analyses presented below, all six *M. annularis* colonies from AG and four colonies of *M. faveolata* from SP were detected as harboring only Clade C *Symbiodinium*. Because the emphasis of this study was on population structure of *Symbiodinium* in Clade B, these ten colonies were excluded from further analyses.

All coral colonies were sampled one to two times per year for genetic analyses between March 2003 and December 2005 by S.C.U.B.A. or snorkel. Additional samples were collected from these tagged colonies for cell count measurements between 2002 and 2006 (up to four total samplings per year). Approximately 10 cm^2^ fragments were removed via hammer and chisel, with care taken to ensure that the same relative position (i.e., unshaded colony tops) was sampled each time. To test for potential sampling effects due to within-colony variation of *Symbiodinium*, tissue was also collected from the sides of colonies in January (NP, Exuma Cays) and March (ADM, Upper Florida Keys) of 2004. Upon collection, fragments were placed in seawater-filled plastic bags and immediately transported to the laboratory in an insulated cooler for processing.

### *Symbiodinium* density and nucleic acids extraction

Coral fragments were processed as follows. Tissue was removed using a recirculating Waterpik (Water Pik Inc.) with 0.45 µm filtered seawater and the “blastate” pulsed for 1–4 s with a Brinkmann Instruments Polytron Kinematica Tissue Homogenizer™ to disperse mucopolysacharides. *Symbiodinium* cell densities were determined following Fitt et al. [35]. Symbiont cells for molecular analyses were isolated from the salt-water “blastate” via centrifugation in 50 mL tubes at 2000–3000 *g* for 5 min, preserved in DMSO Buffer (20% dimethyl sulfoxide and 0.25 M ethylenediaminetetraacetic acid [EDTA] in NaCl-saturated water [36]) and nucleic acids subsequently extracted following Thornhill et al. [31].

### ITS2-PCR and Denaturing Gradient Gel Electrophoresis (DGGE) of *Symbiodinium*

Polymerase chain reaction and denaturing gradient gel electrophoresis (PCR-DGGE) of the ITS2 rDNA was used to discriminate *Symbiodinium* “types” [18]–[19] harbored by the tagged colonies. Following PCR, ITS2 sequences were electrophoresed according to the protocol of Sampayo et al. [37], with the exceptions noted below. Amplified ITS2 fragments were loaded on 20 cm long, 0.75 mm wide, 8% polyacrylamide (37.5∶1 acrylamide/bisacrylamide ratio) gels containing a 45–80% denaturing gradient (100% denaturant contains 7 mol L^−1^ urea and 40% deionized formamide) and electrophoresed at 150 V for 10 h at 60°C on a C.B.S. Scientific™ DGGE-2001 model apparatus. To diagnose symbiont identity, the DGGE fingerprint for each sample was compared to ITS2 standards of known nucleotide sequences. To further validate identifications, all bands in the DGGE-profile were excised, reamplified, and bidirectionally sequenced in 25 of 213 samples using the Genome Lab™ Quick Start Mix (Beckman Coulter) on a Beckman CEQ 8000 Genetic Analysis System (Beckman Coulter) according to the protocol of LaJeunesse [19]. Criteria for band excision and sequencing were as follows: 1) profiles that differed in any way from the ITS2 standards were excised and sequenced to insure they were properly diagnosed, 2) samples were regularly selected at random to insure that no misidentifications due to fragment co-migration had occurred. Chromatograms were checked by comparison to the complement DNA strand in sequencher v4.6 (Gene Codes) and finished sequences aligned manually using macclade v4.06 (Sinauer Associates). Sequences were deposited in GenBank under Accession Nos. GQ268415-GQ268420.

### *Symbiodinium* microsatellite analyses

Three microsatellite loci specific to *Symbiodinium* Clade B (i.e., CA6.38, B7Sym34, and B7Sym36; [38]–[39]) were utilized in this study. Previous work with these loci [38]–[39] and experimental tests using 16 different, clonal *Symbiodinium* Clade B cultures [D.J. Thornhill, Y. Xiang, and S.R. Santos unpublished data] demonstrated that DNA from the coral host is not amplified by these markers and that only a single allele is recovered per Clade B genome since *Symbiodinium* vegetative cells are considered haploid [38], [40]–[42].

Microsatellite amplifications were performed in 10 µL volumes containing 10 mM Tris-HCl (pH 8.3), 50 mM KCl, 0.001% gelatin, 200 µM dNTP, 1.5 mM (CA6.38) or 2.5 mM (B7Sym34/B7Sym36) MgCl_2_, 0.5 U *Taq* polymerase, 0.15 µM WellRED D2, D3 or D4 fluorescent-labeled M-13 primer (Sigma-Proligo), 0.30 µM forward primer, 0.15 µM reverse primer and approximately 10 ng of template DNA. Nineteen nucleotides (5′-CACGACGTTG TAAAACGAC-3′) were added to the 5′ end of reverse primers to allow the incorporation of the M13 fluorescent-labeled primer into PCR products [see 38]. Thermocycling conditions were: initial denaturation 2 min at 94°C; 32 (B7Sym34/B7Sym36) or 40 (CA6.38) cycles of denaturation 94°C for 30 s, annealing 56.0°C (CA6.38) or 57.0°C (B7Sym34/B7Sym36) for 30 s, and extension 72°C for 30 s; followed by a final extension of 5 min at 72°C. Microsatellite allele size determinations were performed on a capillary-based Beckman CEQ 8000 Genetic Analysis System (Beckman Coulter) under the default fragment analysis parameters. Each well contained 4 µL of PCR product, 20 µL sample loading solution (Beckman Coulter) and 0.5 µL 400 bp DNA size ladder (Beckman Coulter). Allele size determinations were conducted twice to verify accurate scoring and are reported according to their true allele size by excluding the 5′ nucleotides of the fluorescent-labeled M13 primers. Successful microsatellite amplifications from all three loci were achieved in a total of 204 of the 213 samples analyzed with DGGE above (97 *M. annularis* and 107 *M. faveolata*). For 9 samples (6 *M. annularis* and 3 *M. faveolata*), amplifications at all three loci were unsuccessful after multiple attempts.

A multilocus genotype was constructed for the *Symbiodinium* Clade B population associated with each *Montastraea* spp. colony using the allele sizes recovered from each locus [22]. In cases were multiple alleles were detected at a single locus, these were interpreted as *Symbiodinium* populations comprised of more than a single Clade B genotype [following 22]. Preliminary analyses revealed that the three loci possessed allelic variation capable of identifying unique *Symbiodinium* haploid genotypes. Along with this, we calculated the probability of identity (PI), or probability that two unrelated host individuals would (by chance alone) have the same multilocus *Symbiodinium* genotype, for both the *M. faveolata* and *M. annularis* datasets in GenAlEx v6.2 [43]. *Symbiodinium* genotypes from each host species were each treated as belonging to a single population. The PIs for *M. faveolata* and *M. annularis* were 2.92×10^−3^ and 4.96×10^−3^, respectively. These values suggest that, at maximum, 2.92 out of 1,000 *M. faveolata* samples and 4.96 out of 1,000 *M. annularis* samples would be mis-identified using these three microsatellites. It should also be noted that these PI values are overestimates because with repetitive sampling of tagged coral colonies, the same *Symbiodinium* genotype is likely to be sampled multiple times in the data set (whereas they are treated as different individuals in the above calculations). Therefore, these three microsatellites have sufficient resolution to differentiate *Symbiodinium* populations from different coral colonies, host species, reefs, and geographic regions with statistical confidence (see Results and Discussion).

To quantify the distribution and relative importance of genetic variation within the examined *Symbiodinium* populations from each host species, analyses of molecular variance (AMOVAs) were performed with the “codom-genotypic” option for haploid data in GenAlEx v6.2 [43]. Total genetic diversity in the *Symbiodinium* populations from each host species was partitioned among groups of populations, among populations within groups, and within populations. Two different models were utilized in the AMOVAs: one based on regional structure and the other on temporal structure. The regional model partitioned variation by geographic region (i.e., Upper Keys vs. Exuma Cays) and among reefs within these regions (i.e., LG, ADM, and AG within the Upper Keys and NP and SP within the Exuma Cays) while the temporal model partitioned variation first by sampling time points and then by reefs within a sampling. It should be noted that the within-colony samples from colony sides collected in January (NP, Exuma Cays) and March (ADM, Upper Florida Keys) of 2004 were not included in the AMOVAs since they represent pseudoreplicates of a single sampling time. Pairwise tests for *Symbiodinium* population differentiation were also conducted by randomizing genotypes between pairs of populations 1) between reefs; and, 2) on a reef through time, using GenAlEx v6.2. Significance of the AMOVAs and pairwise tests were assessed by 10,000 permutations.

## Results

Nearly all *Montastraea* spp. examined from the Florida Keys and Exuma Cays harbored *Symbiodinium* of ITS2 “type” B1 (Table 1). An exception to this pattern was *M. annularis* from Little Grecian Reef, which hosted *Symbiodinium* ITS2 “type” B10. Occasionally, other *Symbiodinium* ITS2 “types” were detected in addition to “types” B1 or B10. These included “types” C3 (*n* = 5 *M. annularis* colonies at ADM), C12 (*n* = 1 *M. faveolata* colony at NP and SP, respectively), and D1a (*n* = 1 *M. faveolata* colony at NP).

**Table 1:** Summary of the *Symbiodinium* ITS2 “types” and multilocus microsatellite genotypes detected in *Montastraea* spp. colonies from the Upper Florida Keys (U.S.) and Exuma Cays (Bahamas).

Sample Information^a^	LG Mf	ADM Mf	AG Mf	NP Mf	SP Mf	LG Ma	ADM Ma	NP Ma	SP Ma	Total
# Colonies	6	6	6	6	2	6	6	6	6	50
Total diversity of ITS2 type(s) detected^b^	B1	B1, C3	B1	B1, C12, D1a	B1, C12	B10	B1, C3	B1	B1, C12	B1, B10, C3, C12, D1a
Total # of detected CA6.38 alleles^b^	1	1	1	1	1	1	1	1	1	3
Total # of detected B7Sym34 alleles^b^	6	3	2	2	1	2	2	2	1	13
Total # of detected B7Sym36 alleles^b^	2	1	1	1	1	1	1	2	2	6
Total # of *Symbiodinum* multilocus microsatellite genotypes per colony over time^b^	1–2	1–3	1–2	1–2	1	1	1–2	1–2	1–2	1–3

a Reef abbreviations provided in Fig. 1; Mf = *M. faveolata*; Ma = *M. annularis*.

b Complete ITS2 and multilocus genotype data available in Tables S1–S2.

Across the *Montastraea* spp., 3, 13, and 6 alleles were identified from microsatellite loci CA6.38, B7Sym34, and B7Sym36, respectively (Table 1). Allele sizes ranged from 115 to 119 bp for CA6.38, 261 to 317 bp for B7Sym34, and 189 to 209 bp for B7Sym36. Within each colony, from one to three microsatellite genotypes were detected over the course of the study (Table 1).

For the *Symbiodinium* Clade B populations of both *M. faveolata* and *M. annularis*, the AMOVAs based on a regional structure model identified significant levels of genetic variation as being partitioned among geographic regions (40–53%) and among reefs within regions (32–47%), with a smaller (but significant) amount of variation (14–15%) among colonies on a reef (Table 2). Under the temporal structure model, none (0.0%) of the genetic variation in the *Symbiodinium* populations was attributable to among sampling time points from either host species (Table 2). Instead, all of the genetic variation was partitioned among reefs (78–83%) or among colonies (17–23%) within sampling time points (Table 2). Specific regional and temporal patterns of genetic variation within and among the *Symbiodinium* Clade B populations of *M. faveolata* and *M. annularis* are detailed in the following subsections.

**Table 2:** Effects of regional and temporal structure on population genetic variation in *Symbiodinium* Clade B populations of *Montastraea* spp. as determined by analysis of molecular variance (AMOVA) of three microsatellite loci.

Host Species and AMOVA Hierarchy	Source of Variation	d.f.	Sum of Squares	Variance Components	% of the Total Variance
*M. faveolata,* Regional Structure	Among regions	1	175.869	3.315	53.19%*
	Among reefs within regions	3	120.515	1.999	32.07%*
	Among colonies within reefs	95	87.275	0.919	14.74%*
	Total	99	383.660		
*M. faveolata,* Temporal Structure	Among sampling time points	3	2.612	0.000	0.00% (NS)
	Among reefs within sampling time points	16	299.548	3.637	78.12%*
	Among colonies within sampling time points	80	81.500	1.019	22.88%*
	Total	99	383.660		
*M. annularis,* Regional Structure	Among regions	1	8.797	0.000	40.47%*
	Among reefs within regions	2	281.403	4.120	46.72%*
	Among colonies within reefs	86	64.833	0.864	13.81%*
	Total	89	355.033		
*M. annularis,* Temporal Structure	Among sampling time points	3	8.797	2.932	0.00% (NS)
	Among reefs within sampling time points	11	281.403	25.582	82.66%*
	Among colonies within sampling time points	75	64.833	0.864	17.34%*
	Total	89	355.033		

* *P*≤0.0001.

(NS) = *P*>0.05.

### Within-colony variation of *Symbiodinium* populations

The *Montastraea faveolata* and *M. annularis* colonies examined here typically harbored *Symbiodinium* ITS2 “type” B1 on both their unshaded tops and shaded sides (Table 3). On the shaded sides of *M. annularis* colonies, this “type” B1 co-occurred with *Symbiodinium* ITS2 “types” C3 (Upper Keys) or C12 (Exuma Cays) (Table 3). Similar intracolonial zonation and intracladal variation of *Symbiodinium* rDNA “types” within *Montastraea* spp. has been documented [e.g., 28]–[30], [32].

**Table 3:** Intracolonial variation in *Symbiodinium* ITS2 “types” and alleles of three microsatellite loci from tagged colonies of *M. faveolata* and *M. annularis* sampled in January (Exuma Cays, Bahamas) and March (Upper Florida Keys, U.S.) of 2004.

Host Species	Reef^a^	Colony	Position	*Symbiodinium* ITS2 type(s)	Locus CA6.38	Locus B7Sym34	Locus B7Sym36
*M. faveolata*	ADM	Colony 1	Top	B1	119	289/293	189
			Side	B1	119	289	189
		Colony 2	Top	B1	119	289	189
			Side	B1	119	293	189
		Colony 3	Top	B1	119	289	189
			Side	B1	119	289	189
		Colony 4	Top	B1	119	293	189
			Side	B1	119	293	189
		Colony 5	Top	B1	119	293	189
			Side	B1	119	289/293	189
		Colony 6	Top	B1	119	289	189
			Side	B1	119	293	189
*M. annularis*	ADM	Colony 1	Top	B1	117	285	205
			Side	B1, C3	117	293	205
		Colony 2	Top	B1	117	285	205
			Side	B1, C3	117	285	205
		Colony 3	Top	B1	117	293	205
			Side	B1, C3	117	293	205
*M. annularis*	NP	Colony 1	Top	B1	115	269	209
			Side	B1^b^, C12	115	269	209
		Colony 2	Top	B1	115	269	209
			Side	B1^b^, C12	115	269	209
		Colony 4	Top	B1	115	269	209
			Side	B1^b^, C12	115	269	209
		Colony 5	Top	B1	115	269	207
			Side	B1, C12	115	269	207

a Reef abbreviations provided in Fig. 1.

b Only ITS2 “type” C12 was identified within the detection threshold of DGGE [31]. However, microsatellite analysis confirmed the presence of ITS2 “type” B1 at sub-DGGE detection levels.

For most samples (*M. faveolata* [92.5%, *n* = 99]; *M. annularis* [98.9%, *n* = 96]), only a single allele was recovered per locus. In the remainder (*M. faveolata* [7.5%, *n* = 8]; *M. annularis* [1.1%, *n* = 1]), two alleles were detected at either B7Sym34 or B7Sym36. Thus, the presence of more than a single allele at these loci is indicative of (at least) two *Symbiodinium* genotypes within these particular samples.

A single multilocus genotype dominated the *Symbiodinium* Clade B populations on both the tops and sides of *Montastraea* spp. colonies from the Bahamas (Table 3). Likewise, the majority (*n* = 7 of 9; Table 3) of colonies examined from the Upper Florida Keys also harbored populations along their tops and sides comprised of a single *Symbiodinium* genotype. However, five colonies (i.e., four *M. faveolata* and one *M. annularis*) from Admiral Reef in the Florida Keys possessed differing, or two, alleles at one locus (i.e., B7Sym34) on their tops versus sides (Table 3). This suggests that while the *Symbiodinium* Clade B populations of *Montastraea* spp. are largely homogeneous, there may occasionally be heterogeneity across an individual colony.

### Between-colony variation of *Symbiodinium* populations on a reef

**Figure 2:**
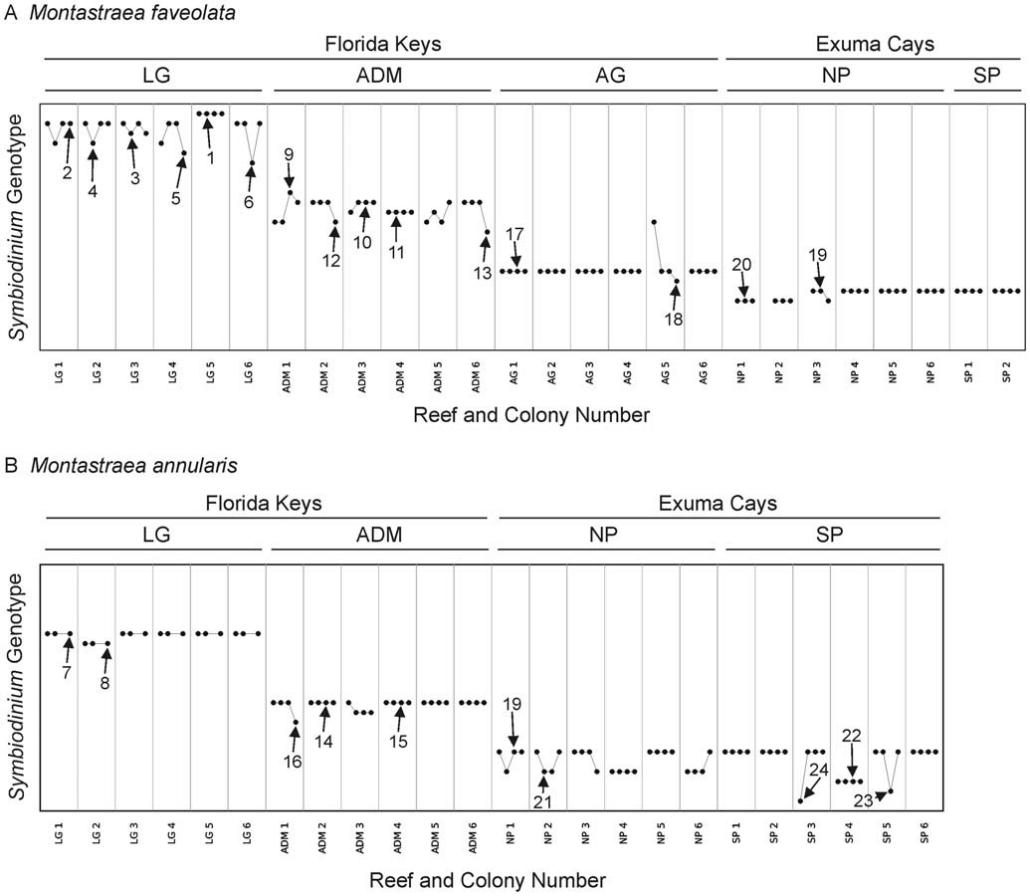
Categorical plots of *Symbiodinium* Clade B multilocus genotypes within *Montastraea* spp. colonies over time. *Symbiodinium* Clade B multilocus genotypes detected within specific colonies of the corals *Montastraea faveolata* (A) and *M. annularis* (B) from the Upper Florida Keys (U.S.) and Exuma Cays (Bahamas). On the y-axes, each unique *Symbiodinium* genotype is assigned a specific vertical position. Arrows and numbers indicate the specific *Symbiodinium* multilocus microsatellite genotype; numbers correspond to genotype numbers presented in Table 4. The x-axes depict each of the tagged colonies (*n* = 50) tracked during this study. Four data points are presented for each colony, corresponding to genotypes detected during each of the four sampling dates (left to right; Florida Keys: March 2003, March 2004, Sept. 2005 and Dec. 2005; Exuma Cays: Jan. 2003, Jan. 2004, Sept. 2005, Nov. 2005). Colonies with only three data points indicate missing data for a particular sampling period. Stability of a *Symbiodinium* population within a coral colony is depicted as a straight, horizontal line whereas changes in the symbiont genotype detected are indicated as a vertical shift between sampling periods. The reef and region for each colony is noted above the graph (reef abbreviations correspond to Fig. 1).

Low genotypic diversity generally characterized the *Symbiodinium* Clade B populations associated with *Montastraea* spp. on a reef (Table 4). This is evident by the AMOVAs, where a relatively low proportion of genetic variation was partitioned among colonies on a reef (14.74% for *M. faveolata*, 13.81% for *M. annularis*; Table 2). For *M. faveolata*, between one (e.g., South Perry Reef, Exuma Cays) and six (e.g., Little Grecian Reef, Upper Florida Keys) *Symbiodinium* multilocus genotypes were identified per reef (Table 4). A similar range (i.e., 2–4) of symbiont genotypes was also recovered from *M. annularis* (Table 4). In most cases, these *Symbiodinium* genotypes were not uniformly distributed across colonies and/or through time. Instead, individual colonies of both species predominantly associated with only one (or more rarely, two to three in the case of *M. faveolata*) of these *Symbiodinium* Clade B genotypes during all sampling time points (Fig. 2, Tables S1–S2). When a differing genotype was recovered from a colony, this was typically at a single time point during the approximately three-year study period (Fig. 2, Tables S1–S2).

**Table 4:** Relative frequencies and distributions of *Symbiodinium* Clade B ITS2 “types” and multilocus microsatellite genotypes in tagged colonies of *M. faveolata* and *M. annularis* collected between 2003 and 2005 in the Upper Florida Keys (U.S.) and Exuma Cays (Bahamas).

Genotype #^a^	ITS2 “type”^b^	Locus CA6.38	Locus B7Sym34	Locus B7Sym36	LG Mf^c^	LG Ma^c^	ADM Mf^c^	ADM Ma^c^	AG Mf^c^	NP Mf^c^	NP Ma^c^	SP Mfc,d	SP Ma^c^
1	B1	117	265	201	0.167	-	-	-	-	-	-	-	-
2	B1	117	305	189	0.542	-	-	-	-	-	-	-	-
3	B1	117	309	189	0.083	-	-	-	-	-	-	-	-
4	B1	117	313	189	0.125	-	-	-	-	-	-	-	-
5	B1	117	305/313	189	0.042	-	-	-	-	-	-	-	-
6	B1	117	317	189	0.042	-	-	-	-	-	-	-	-
7	B10	119	265	203	-	0.833	-	-	-	-	-	-	-
8	B10	119	273	203	-	0.167	-	-	-	-	-	-	-
9	B1	119	285	189	-	-	0.033	-	-	-	-	-	-
10	B1	119	289	189	-	-	0.433	-	-	-	-	-	-
11	B1	119	293	189	-	-	0.300	-	0.042	-	-	-	-
12	B1	119	289/293	189	-	-	0.200	-	-	-	-	-	-
13	B1	119	297	189	-	-	0.033	-	-	-	-	-	-
14	B1	117	285	205	-	-	-	0.778	-	-	-	-	-
15	B1	117	293	205	-	-	-	0.185	-	-	-	-	-
16	B1	117	285/293	205	-	-	-	0.037	-	-	-	-	-
17	B1	119	261	189	-	-	-	-	0.917	-	-	-	-
18	B1	119	261/293	189	-	-	-	-	0.042	-	-	-	-
19	B1	115	269	207	-	-	-	-	-	0.667	0.464	1.000	0.750
20	B1	115	273	207	-	-	-	-	-	0.333	0.036	-	-
21	B1	115	269	209	-	-	-	-	-	-	0.500	-	-
22	B1	115	269	203	-	-	-	-	-	-	-	-	0.167
23	B1	115	269	205	-	-	-	-	-	-	-	-	0.042
24	B1	115	265	207	-	-	-	-	-	-	-	-	0.042
n					24	18	30	27	24	21	28	8	24

a Multilocus genotype numbers correspond to those presented in Fig. 2.

b ITS2 data only include detected Clade B “types”. Complete ITS2 information provided in Tables S1–S2.

c Reef abbreviations provided in Fig. 1; Mf = *M. faveolata*; Ma = *M. annularis*.

d *Montastraea faveolata* colonies 3 through 6 from South Perry reef harbored only *Symbiodinium* ITS2 “type” C12. Thus, these colonies were not analyzed with the three *Symbiodinium* Clade B-specific microsatellite markers. For this reason, these colonies are not included here.

In the Upper Florida Keys, the *Symbiodinium* Clade B populations harbored by *M. faveolata* and *M. annularis* were distinct, with no genotypes being common between the two species (Table 4). Given this, all interspecies pairwise tests of *Symbiodinium* population differentiation between the *Montastraea* spp. on these reefs were significant (*P*<0.005). In contrast, two multilocus genotypes were shared by *M. faveolata* and *M. annularis* from the Exuma Cays (Table 4). Here, pairwise tests of population differentiation were significant between *M. faveolata* and *M. annularis* on North Norman's Patch Reef (*P*<0.005); however, similar comparisons between South Perry *M. faveolata* and *M. annularis* were not significant (*P*>0.005).

### *Symbiodinium* population variation between reefs and regions

The *Symbiodinium* Clade B populations of *Montastraea* spp. in the Upper Florida Keys exhibited strong endemism to reefs (Tables 2,4). Specifically, 12 of 13 genotypes harbored by *M. faveolata* were site-specific. The single outlier to this pattern was a genotype recovered on both Admiral (9 of 30 samples) and Alligator (1 of 23 samples) Reefs. In the case of *M. annularis*, all genotypes were site-specific. Thus, all pairwise tests of between-reef *Symbiodinium* population differentiation by species were significant (*P*<0.005) in the Upper Florida Keys.

In contrast, the *Symbiodinium* Clade B populations of *Montastraea* spp. in the Exuma Cays were less structured. Although two and three *Symbiodinium* genotypes associated with *M. annularis* were site-specific to North Norman's Patch and South Perry Reefs, respectively, one genotype was common to colonies of this species at both sites (Table 4). This particular *Symbiodinium* genotype was also the most frequently recovered from *M. faveolata* at North Norman's Patch and South Perry Reefs (Table 4). This led to pairwise tests of between-reef population differentiation being significant (*P*<0.005) for *Symbiodinium* Clade B harbored by *M. annularis*, but not for those of *M. faveolata*.

Regionally, no *Symbiodinium* Clade B genotypes were common between the Upper Florida Keys and the Exuma Cays (Table 4). Thus, all regional pairwise tests for population differentiation were significant (*P*<0.005).

### *Symbiodinium* population stability through time and a coral bleaching episode

This study complements the multi-year temporal tracking of *Symbiodinium* ITS2 “types” associated with *Montastraea* spp. [31] by extending it to the population level for symbionts from Clade B. As previously reported, colonies generally harbored the same ITS2 “type” over time (Tables S1–S2), with all *M. faveolata* and most *M. annularis* associated with *Symbiodinium* ITS2 “type” B1 (the exception being *M. annularis* from Little Grecian Reef, which harbored ITS2 “type” B10). Likewise, the same *Symbiodinium* Clade B multilocus genotype was maintained by the majority of colonies throughout the study duration ((*M. faveolata* [57.7%, 15 of 26 colonies]; *M. annularis* [75.0%, 18 of 24 colonies]); Fig. 2, Tables S1–S2). In the remaining colonies with two or three symbiont genotypes (*M. faveolata* with two [*n* = 7] or three genotypes [*n* = 4]; *M. annularis* with two genotypes [*n* = 6]), these tended to temporally alternate and/or were maintained as a mixed population (Fig. 2). As a consequence of this stability within a colony and population variation between colonies and reefs, AMOVAs revealed that none of the genetic variation was attributable to among sampling time points (Table 2). Along with this, pairwise tests found no statistically significant change in the *Symbiodinium* Clade B populations from any of the sampled *Montastraea* spp. colonies occurred over time (*P*>0.05).

**Figure 3:**
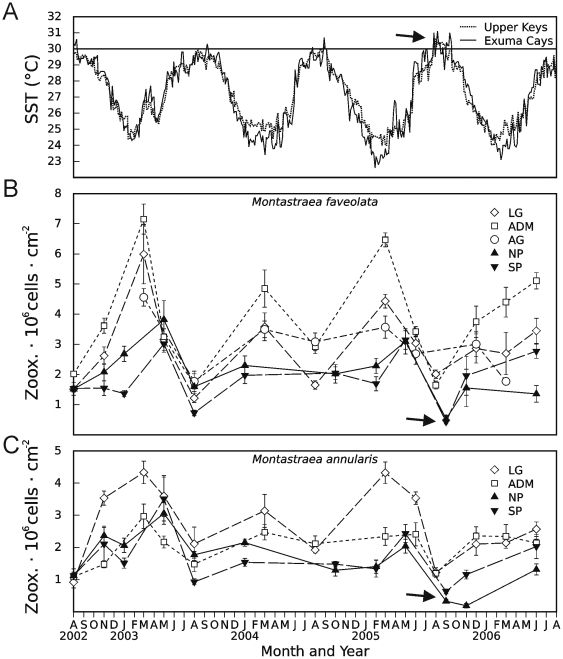
Sea-surface temperatures and *Symbiodinium* cell densities from *Montastraea* spp. corals. Nocturnal sea-surface temperatures (A) in the Upper Florida Keys (U.S.) and Exuma Cays (Bahamas) between August 2002 and August 2006. Solid line demarcates the approximate ‘high-temperature bleaching threshold’ of 30°C, while an arrow notes the increased frequency of temperatures above 30°C in 2005 relative to preceding years. *Symbiodinium* cell densities in tagged colonies of *Montastraea faveolata* (B) and *M. annularis* (C) from the five collection sites, three in the Upper Florida Keys (white markers) and two in Exuma Cays (black markers). Cell density data are expressed as means±SE. Arrows denote decreased symbiont cell densities correlated with the high-temperature bleaching event in the Exuma Cays.

**Figure 4:**
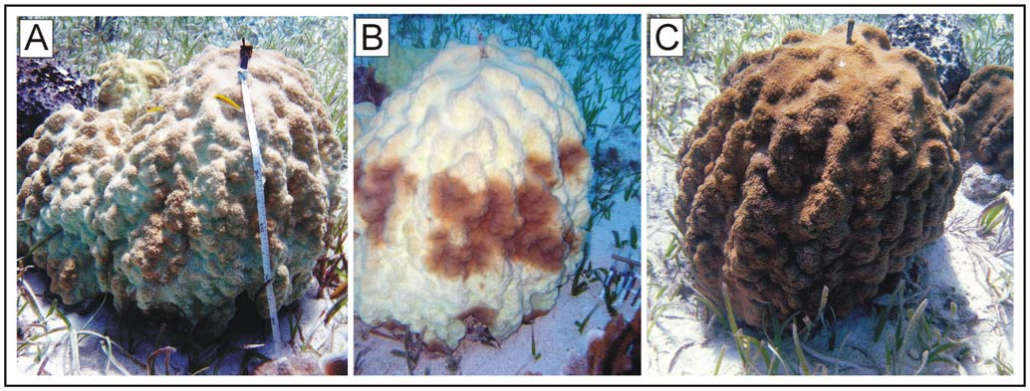
Photographs of a bleached *Montastraea faveolata* colony during and following the 2005 high-temperature bleaching event. Photographs of a single colony of *M. faveolata* from Norman's Patch reef (Exuma Cays, Bahamas) during the bleaching event in September 2005 (A), following the bleaching event in November 2005 (B), and following recovery in June 2006 (C). Images have been histogram equalized to reduce underexposure from surrounding sea-water. Photo credits: Dustin W. Kemp and Jennifer McCabe-Reynolds.

The temporal sampling scheme of this study also provided an opportunity to observe how *Symbiodinium* Clade B populations respond to a temperature-induced bleaching episode. During the summer of 2005, *Montastraea* spp. in the Exuma Cays were exposed to temperatures >30°C for an extended period of time, with conditions exceeding those in preceding years (Fig. 3). As a result, extensive bleaching was recorded from many scleractinian species at both North Norman's Patch and South Perry Reefs (Fig. 4, [33]). Visible paling of the colonies sampled for this study was accompanied by a concomitant decrease in the density of *Symbiodinium* to levels at, or below, 5×10^5^ cells · cm^−2^ of host tissue (Fig. 3). Essentially, this bleaching event reduced *Symbiodinium* densities well below those observed during the normal seasonal fluctuations of symbionts in *Montastraea* spp. [35].

Within this context, little variation was observed in the ITS2-type of *Symbiodinium* associated with either *M. faveolata* or *M. annularis* over the course of the 2005 bleaching episode, with colonies continuing to maintain symbioses with either *Symbiodinium* ITS2 “types” B1 or B10 (Tables S1–S2; note the detection of ITS2 “type” D1a in one colony of *M. faveolata*) [31]. Similarly, this event had no detectable effect on the *Symbiodinium* Clade B populations of these *Montastraea* spp. colonies (Table 2, Fig. 2). In most cases, a given host harbored the same *Symbiodinium* multilocus genotype before, during, and following the event. Furthermore, while four colonies (i.e., one *M. faveolata* and three *M. annularis*) did exhibit compositional changes in their populations through the event, the recovered genotypes were consistent with those encountered at previous time points from within the same (or other Exuma Cays') individuals (Table 2, Fig. 2). Thus, no statistically significant change in the *Symbiodinium* Clade B populations from any of the colonies was observed as a result of the bleaching episode (*P*>0.05).

## Discussion

This study is the first fine-scale comparative and temporal analysis of genetic diversity and population structure for *Symbiodinium* associated with two ecologically dominant reef-building scleractinian corals in the Caribbean, *Montastraea faveolata* and *M. annularis*. Here, microsatellite markers revealed *Montastraea* spp. harboring *Symbiodinium* Clade B populations that were generally endemic to a reef, specific to a given host species, and temporally stable through both seasonal change and environmental perturbation in the form of a high temperature-induced bleaching event. The significance of these results is discussed below.

### Patterns and processes of genetic structure between *Symbiodinium* populations

Strong population structure ranging from between-species comparisons on the same reef to within-species surveys at geographic sites separated by tens to hundreds of kilometers characterizes the *Symbiodinium* “type” B1 populations of *Montastraea* spp. in the Upper Florida Keys and Bahamas. While an identical *Symbiodinium* multilocus genotype inhabits the majority of colonies of a host species on a given reef, the sharing of genotypes between sites separated by ∼12.5 km or more is nearly non-existent. These findings are consistent with reports of strong differentiation between *Symbiodinium* “type” B1 populations from other Caribbean host species, such as the gorgonians *Pseudopterogorgia elisabethae* [22] and *Gorgonia ventalina* [23]–[24] in the Bahamas and Florida Keys, respectively. Similar patterns have also been documented in another *Symbiodinium* Clade and oceanic basin. Specifically, the *Symbiodinium* Clade C populations of the alcyonacean coral *Sinularia flexibilis* are significantly structured along the Great Barrier Reef in the Pacific Ocean at scales from ∼16–1,360 km [25]. Given this, a potentially ubiquitous feature of *Symbiodinium* populations is high levels of structure over spatial distances as small as 10's of km.

In contrast to the above, *Symbiodinium* Clade C populations hosted by the scleractinian *Pocillopora meandrina* were reported to have no structure across ∼200 km in the central and western Pacific Ocean [26]. One explanation for this could relate to Pacific current systems facilitating dispersal and connectivity between populations. Alternatively, differences in a host's mode of *Symbiodinium* acquisition are a potential driver for these patterns [44]. Specifically, although *P. elisabethae*, *G. ventalina*, *S. flexibilis*, *M. faveolata*, and *M. annularis* acquire their symbionts horizontally from the environment [22], [24]–[25], [27], [34], *P. meandrina* transmits *Symbiodinium* vertically from maternal parent to offspring [26]. Thus, since vertically transmitted *Symbiodinium* disperse in concert with their host's progeny, this may convey greater dispersal capability and higher levels of connectivity relative to symbionts horizontally transferred through the environment.

One interpretation for the low diversity of the examined *Montastraea* spp. *Symbiodinium* Clade B populations on a reef is that the coral hosts are propagating primarily via asexual reproduction (i.e., fragmentation) and that the same genetic individual (i.e., genet), harboring an identical *Symbiodinium* genotype, was independently sampled multiple times. Such a scenario would lead to the perception that a single *Symbiodinium* genotype dominates that particular host species on a reef. However, this hypothesis seems unlikely for two reasons. Firstly, sexual reproduction is the dominant reproductive mode in *Montastraea* spp. [27] and previous studies demonstrated only low levels of clonality in *M. faveolata* and *M. annularis* [9], [45]. Secondly, when they occur, multiple colony genets are generally in close proximity to one another (i.e., 0.15 to 6.94 m apart) in, for example, *M. annularis* [45]. Given that our sampling scheme utilized spatially separated colonies (4 to 50 m apart) and assuming levels of clonality consistent with previous reports [9], [45], host clonality should have a minimal impact on the dataset since less than two clonal colonies per host species per reef would have been tagged and sampled during the course of this study.

Overall, the available data suggest dispersal of *Symbiodinium* as environmental pools is generally limited. This is particularly relevant since a majority of hosts acquire their symbiont(s) anew from such pools at each generation. For example, approximately 81 genera in the broadcast spawning scleractinian corals alone depend on this mode of *Symbiodinium* infection [46]. Thus, although it has been established that these symbionts are present in the environment [47]–[54] and aposymbiotic hosts quickly acquire them when exposed to natural seawater [e.g., 55]–[56], analyses of within-host populations imply that they do not disperse widely since most are endemic to a single reef (see Results and references above). Like many dinoflagellates, *Symbiodinium* is capable of independent dispersal through the water column [57]–[59] as they alternate between a vegetative and flagellated/motile state [60]–[61]. However, *Symbiodinium* motility is governed by light conditions and cells swim just 3–10 m per vegetative and dividing period [61]. Such behaviors have the potential to retain these symbionts within localized populations (i.e., a given reef) and contribute to the geographic isolation reported here and elsewhere.

An alternative hypothesis for the strong structure observed in *Symbiodinium* populations is that while genotypes are fully capable of dispersing between reefs, migrants are rarely successful at establishing a new symbiosis upon arrival due to competition with the resident/endemic genotype. Invertebrates harboring *Symbiodinium* constantly expel symbiont cells as a means of density regulation [58], [62]–[65]; this would result in the resident/endemic genotype for that particular reef and/or host species occurring at higher densities than that of immigrants in the environmental infection pool. Under such a scenario, an aposymbiotic host would have a higher probability of acquiring the numerically dominant *Symbiodinium* genotype present in that environment, which in this case is from the resident/endemic population. Thus, the density-dependent feedback loop created by these cycles of expulsion and reacquisition could severely limit the opportunity for novel *Symbiodinium* genotypes to penetrate and homogenize the established population on a reef.

### *Symbiodinium* specificity within and among host species

At the population level, *M. faveolata* and *M. annularis* in the Upper Florida Keys harbored distinct *Symbiodinium* “type” B1 genotypes (Table 4). This is particularly noteworthy since both 1) acquire their *Symbiodinium* from the environment at each generation, and; 2) occur immediately adjacent to one another on the same reef. Similar patterns of *Symbiodinium* population partitioning in sympatric and congeneric host species have been reported for *Pseudopterogorgia* spp., *G. ventalina* [34] and *Pocillopora* spp. [26], with this phenomenon attributed to fine-scale specificity between partners. Thus, while *Montastraea* spp. are among the most flexible cnidarians known when it comes to their symbiotic associations (see Introduction), specific host-symbiont pairings are clearly evident in the *Symbiodinium* populations of these scleractinian corals from the Upper Florida Keys.

In contrast, two *Symbiodinium* genotypes were common between the *Montastraea* spp. on Bahamian reefs (Table 4). Although it is possible that additional polymorphic microsatellite markers might differentiate these *Symbiodinium* populations, one hypothesis for this regional variability in pairings is that host-*Symbiodinium* specificity is influenced by the degree of hybridization between these coral species. Based on molecular genetic and morphological evidence, Fukami et al. [66] reported higher frequencies of introgression between *M. faveolata* and *M. annularis* in the Bahamas relative to Panama, suggesting the existence of a geographic gradient in hybridization between closely related *Montastraea* across the Caribbean [see also 67]. Given this, it is conceivable that *M. faveolata* and *M. annularis* hybridize less frequently in the Upper Florida Keys compared to the Exuma Cays and anecdotal observations of *Montastraea* spp. coralite morphology support this conjecture (D.J. Thornhill and W.K. Fitt, personal observations). Thus, *Montastraea* spp.-*Symbiodinium* specificity at the population level appears to be tracking host genetics and morphology, with introgression in the Bahamas breaking down the mechanism(s) leading to specificity and potential co-evolution between the symbiotic partners. Future studies might test this hypothesis by comparing the fine-scale molecular genetics of these hosts in relation to their *Symbiodinium* populations.

### Temporal and stress stability of *Symbiodinium* populations

Most of the *Montastraea* spp. colonies examined here maintained the same *Symbiodinium* Clade B genotype(s) over a three-year duration. In cases where change was observed, this was typically a brief (i.e., between two sampling time points) transition between genotypes previously recovered from that colony and/or endemic to that particular reef (Table 4, Fig. 2). While the sampling design of this study cannot identify the specific mechanism(s) behind this variability, potential reasons include spatial heterogeneity of genotypes within the *Symbiodinium* population of a colony (Table 3), shuffling in the relative densities of genotypes within a population [e.g., 68]–[69], and/or switching through the acquisition of genotypes from the external environment [e.g., 70]. Notably, no more than two *Symbiodinium* Clade B genotypes were simultaneously recovered from any *Montastraea* spp. colony over the study period (Table 3, Tables S1–S2), which is consistent with previous population-level analyses or microsatellite screening from Caribbean cnidarians harboring this Clade [22]–[24], [39], [71]. Such a pattern suggests within-host competition may be occurring between closely related *Symbiodinium* [72], with a stable maximum of only two Clade B genotypes co-occupying a single host at a given time. This contrasts sharply with comparable analyses of *Symbiodinium* Clade C populations, where individual hosts appear capable of harboring >2 (and up to 11) genotypes simultaneously [25]–[26]. Further work is needed to determine whether these variable levels of within-host population heterogeneity are confined to the examined cnidarian species or are general and specific characteristics of Clades B and C, respectively, as well as if the other *Symbiodinium* Clades follow these precedents.

In addition to temporal stability, the *Symbiodinium* Clade B populations of *Montastraea* spp. exhibited resilience in the face of physiological and/or environmental perturbation (Figs. 2–3). Specifically, the 2005 bleaching event in the Bahamas did not induce any statistically significant change in the genotypic composition of populations harbored by the examined colonies and only limited change at the ITS2-rDNA level (Fig. 2, Tables S1–S2). Thus, while bleaching has been postulated to drive acclimatization through changes in host-*Symbiodinium* associations [e.g., 73]–[74], no support for this hypothesis is evident in these data from *M. faveolata*, and *M. annularis*. Instead, severe bleaching events often result in substantial coral death through differential mortality [e.g., 75], with concomitant reductions in the biodiversity of an affected reef [12 and references within]. Because populations represent the fundamental unit of evolution and due to the population-level endemism and fine-scale specificity of *Symbiodinium* seen here and in other studies [22]–[26], [34], the potential loss of host and symbiont diversity from coral reefs via bleaching events may ultimately compromise the adaptive capacity and long-term persistence of many symbiotic invertebrates.

## Supporting Information

Table S1 Temporal patterns of *Symbiodinium* ITS2 “type(s)” and multilocus microsatellite genotype(s) from tagged *M. faveolata* colonies sampled between 2003 and 2005 in the Upper Florida Keys and Exuma Cays.
(0.07 MB DOC)

Table S2 Temporal patterns of *Symbiodinium* ITS2 “type(s)” and multilocus microsatellite genotype(s) from tagged *M. annularis* colonies sampled between 2003 and 2005 in the Upper Florida Keys and Exuma Cays.
(0.06 MB DOC)
