# Supplementary material for: Reef Endemism, Host Specificity and Temporal Stability in Populations of Symbiotic Dinoflagellates from Two Ecologically Dominant Caribbean Corals
Source: PLoS One. 2009 Jul 15;4(7):e6262. doi: 10.1371/journal.pone.0006262 (PMC2706050; doi:10.1371/journal.pone.0006262)
Supplement: Table S1 — Temporal patterns of Symbiodinium ITS2 “type(s)” and multilocus microsatellite genotype(s) from tagged M. faveolata colonies sampled between 2003 and 2005 in the Upper Florida Keys and Exuma Cays. (0.07 MB DOC) [file pone.0006262.s001.doc]

**Table S1:** Temporal patterns of *Symbiodinium* ITS2 “type(s)” and multilocus microsatellite genotype(s) from tagged *M. faveolata* colonies sampled between 2003 and 2005 in the Upper Florida Keys (U.S.) and Exuma Cays (Bahamas).

| **Region** | **Reef^a^** | **Colony #** | **Mar-03^b^** | **Mar-04^b^** | **Sep-05^b^** | **Dec-05^b^** | **Total # of *Symbiodinum* multilocus microsatellite genotypes over time** |
| --- | --- | --- | --- | --- | --- | --- | --- |
| Upper Florida Keys | LG | 1 | B1; 117; 305; 189 | B1; 117; 313; 189 | B1; 117; 305; 189 | B1; 117; 305; 189^c^ | 2 |
|  |  | 2 | B1; 117; 305; 189 | B1; 117; 313; 189 | B1; 117; 305; 189 | B1; 117; 305; 189^c^ | 2 |
|  |  | 3 | B1; 117; 305; 189 | B1; 117; 309; 189 | B1; 117; 305; 189 | B1; 117; 309; 189^c^ | 2 |
|  |  | 4 | B1; 117; 313; 189 | B1; 117; 305; 189 | B1; 117; 305; 189 | B1; 117; 305/313; 189^c^ | 2^d^ |
|  |  | 5 | B1; 117; 265; 201 | B1; 117; 265; 201 | B1; 117; 265; 201 | B1; 117; 265; 201^c^ | 1 |
|  |  | 6 | B1; 117; 305; 189 | B1; 117; 305; 189 | B1; 117; 317; 189 | B1; 117; 305; 189^c^ | 2 |
|  | ADM | 1 | B1; 119; 289/293; 189 | B1; 119; 289/293; 189 | B1; 119; 285; 189 | B1; 119; 289; 189 | 3^d,e^ |
|  |  | 2 | B1; 119; 289; 189 | B1; 119; 289; 189 | B1; 119; 289; 189 | B1; 119; 289/293; 189 | 2^d,e^ |
|  |  | 3 | B1; 119; 293; 189 | B1; 119; 289; 189 | B1; 119; 289; 189 | B1; 119; 289; 189 | 2^e^ |
|  |  | 4 | B1; 119; 293; 189 | B1; 119; 293; 189 | B1; 119; 293; 189 | B1; 119; 293; 189 | 1^e^ |
|  |  | 5 | B1; 119; 289/293; 189 | B1; 119; 293; 189 | B1; 119; 289/293; 189 | B1; 119; 289; 189 | 2^d,e^ |
|  |  | 6 | B1; 119; 289; 189 | B1; 119; 289; 189 | B1; 119; 289; 189 | B1; 119; 297; 189 | 3^e^ |
|  | AG | 1 | B1; 119; 261; 189 | B1; 119; 261; 189 | B1; 119; 261; 189 | B1; 119; 261; 189 | 1 |
|  |  | 2 | B1; 119; 261; 189 | B1; 119; 261; 189 | B1; 119; 261; 189 | B1; 119; 261; 189 | 1 |
|  |  | 3 | B1; 119; 261; 189 | B1; 119; 261; 189 | B1; 119; 261; 189 | B1; 119; 261; 189 | 1 |
|  |  | 4 | B1; 119; 261; 189 | B1; 119; 261; 189 | B1; 119; 261; 189 | B1; 119; 261; 189 | 1 |
|  |  | 5 | B1; 119; 293; 189 | B1; 119; 261; 189 | B1; 119; 261; 189 | B1; 119; 261/293; 189 | 2^d^ |
|  |  | 6 | B1; 119; 261; 189 | B1; 119; 261; 189 | B1; 119; 261; 189 | B1; 119; 261; 189 | 1 |
|  |  |  | **Jan-03^b^** | **Jan-04^b^** | **Sep-05^b^** | **Nov-05^b^** |  |
| Exuma Cays | NP | 1 | B1/C12; ND; ND; ND | B1; 115; 273; 207 | B1; 115; 273; 207 | B1; 115; 273; 207 | 1 |
|  |  | 2 | B1; 115; ND; 207 | B1; 115; 273; 207 | B1; 115; 273; 207 | B1; 115; 273; 207 | 1 |
|  |  | 3 | B1; ND; ND; ND | B1; 115; 269; 207 | B1; 115; 269; 207 | B1; 115; 273; 207 | 2 |
|  |  | 4 | B1; 115; 269; 207 | B1; 115; 269; 207 | B1; 115; 269; 207 | B1/D1a; 115; 269; 207 | 1 |
|  |  | 5 | B1; 115; 269; 207 | B1; 115; 269; 207 | B1; 115; 269; 207 | B1; 115; 269; 207 | 1 |
|  |  | 6 | B1; 115; 269; 207 | B1; 115; 269; 207 | B1; 115; 269; 207 | B1; 115; 269; 207 | 1 |
|  | SP^f^ | 1 | B1; 115; 269; 207 | B1; 115; 269; 207 | B1/C12; 115; 269; 207 | B1/C12; 115; 269; 207 | 1 |
|  |  | 2 | B1; 115; 269; 207 | B1; 115; 269; 207 | B1; 115; 269; 207 | B1; 115; 269; 207 | 1 |

^a^: Reef abbreviations provided in Fig. 1.

^b^: Data listed as ITS2 “type(s)”, followed by genotype(s) defined as allele sizes for loci CA6.38, B7Sym34, and B7Sym36, respectively. ND (no data) indicates that these alleles from this microsatellite locus failed to amplify successfully after multiple attempts with this sample.

^c^: Sample collected in March 2006 instead of December 2005.

^d^: In this colony, a total of two different allele sizes were detected at loci B7Sym34. At different time points, these alleles were detected both autonomously and together in the same sample (e.g., 305, 313, and 305/313). Because *Symbiodinium* is a haploid organism [39,40-42], detection of two alleles in one sample is indicative of a mixed population of two symbiont genotypes rather than a heterozygous genotype. Therefore, this colony was scored as harboring two genotypes (e.g., 305 and 313) rather than three (e.g., 305, 313, and 305/313).

^e^: Includes data from the colony sides presented in Table 2.

^f^: *Montastraea faveolata* colonies 3 through 6 from South Perry reef harbored only *Symbiodinium* ITS2 “type” C12. Thus, these colonies were not analyzed with the three *Symbiodinium* Clade B-specific microsatellite markers. For this reason, these colonies are not included here.
