# Supplementary material for: Reef Endemism, Host Specificity and Temporal Stability in Populations of Symbiotic Dinoflagellates from Two Ecologically Dominant Caribbean Corals
Source: PLoS One. 2009 Jul 15;4(7):e6262. doi: 10.1371/journal.pone.0006262 (PMC2706050; doi:10.1371/journal.pone.0006262)
Supplement: Table S2 — Temporal patterns of Symbiodinium ITS2 “type(s)” and multilocus microsatellite genotype(s) from tagged M. annularis colonies sampled between 2003 and 2005 in the Upper Florida Keys and Exuma Cays. (0.06 MB DOC) [file pone.0006262.s002.doc]

**Table S2:** Temporal patterns of *Symbiodinium* ITS2 “type(s)” and multilocus microsatellite genotype(s) from tagged *M. annularis* colonies sampled between 2003 and 2005 in the Upper Florida Keys (U.S.) and Exuma Cays (Bahamas).

| **Region** | **Reef^a^** | **Colony #** | **Mar-03^b^** | **Mar-04^b^** | **Sep-05^b^** | **Dec-05^b^** | **Total # of *Symbiodinum* multilocus microsatellite genotypes over time** |
| --- | --- | --- | --- | --- | --- | --- | --- |
| Upper Florida Keys | LG | 1 | B10; 119; 265; 203 | B10; 119; 265; 203 | B10; ND; ND; ND | B10; 119; 265; 203 | 1 |
|  |  | 2 | B10; 119; 273; 203 | B10; 119; 273; 203 | B10; ND; ND; ND | B10; 119; 273; 203 | 1 |
|  |  | 3 | B10; 119; 265; 203 | B10; 119; 265; 203 | B10; ND; ND; ND | B10; 119; 265; 203 | 1 |
|  |  | 4 | B10; 119; 265; 203 | B10; 119; 265; 203 | B10; ND; ND; ND | B10; 119; 265; 203 | 1 |
|  |  | 5 | B10; 119; 265; 203 | B10; 119; 265; 203 | B10; ND; ND; ND | B10; 119; 265; 203 | 1 |
|  |  | 6 | B10; 119; 265; 203 | B10; 119; 265; 203 | B10; ND; ND; ND | B10; 119; 265; 203 | 1 |
|  | ADM | 1 | B1; 117; 285; 205^c^ | B1; 117; 285; 205 | B1/C3; 117; 285; 205 | B1; 117; 285/293; 205 | 2^d,e^ |
|  |  | 2 | B1^f^/C3; 117; 285; 205^c^ | B1; 117; 285; 205 | B1; 117; 285; 205 | B1; 117; 285; 205 | 1^e^ |
|  |  | 3 | B1; 117; 285; 205^c^ | B1; 117; 293; 205 | B1/C3; 117; 293; 205 | B1; 117; 293; 205 | 2^e^ |
|  |  | 4 | B1; 117; 285; 205^c^ | B1; 117; 285; 205 | B1; 117; 285; 205 | B1; 117; 285; 205 | 1 |
|  |  | 5 | B1/C3; 117; 285; 205^c^ | B1; 117; 285; 205 | B1; 117; 285; 205 | B1; 117; 285; 205 | 1 |
|  |  | 6 | B1/C3; 117; 285; 205^c^ | B1; 117; 285; 205 | B1; 117; 285; 205 | B1; 117; 285; 205 | 1 |
|  |  |  | **Jan-03^b^** | **Jan-04^b^** | **Sep-05^b^** | **Nov-05^b^** |  |
| Exuma Cays | NP | 1 | B1; 115; 269; 207 | B1; 115; 269; 209 | B1; 115; 269; 207 | B1; 115; 269; 207 | 2^e^ |
|  |  | 2 | B1; 115; 269; 207 | B1; 115; 269; 209 | B1; 115; 269; 209 | B1; 115; 269; 207 | 2^e^ |
|  |  | 3 | B1; 115; 269; 207 | B1; 115; 269; 207 | B1; 115; 269; 207 | B1; 115; 269; 209 | 2 |
|  |  | 4 | B1; 115; 269; 209 | B1; 115; 269; 209 | B1; 115; 269; 209 | B1; 115; 269; 209 | 1^e^ |
|  |  | 5 | B1; 115; 273; 207 | B1; 115; 269; 207 | B1; 115; 269; 207 | B1; 115; 269; 207 | 2^e^ |
|  |  | 6 | B1; 115; 269; 209 | B1; 115; 269; 209 | B1; 115; 269; 209 | B1; 115; 269; 207 | 2 |
|  | SP | 1 | B1; 115; 269; 207 | B1; 115; 269; 207 | B1; 115; 269; 207 | B1; 115; 269; 207 | 1 |
|  |  | 2 | B1; 115; 269; 207 | B1; 115; 269; 207 | B1; 115; 269; 207 | B1; 115; 269; 207 | 1 |
|  |  | 3 | B1; 115; 265; 207 | B1; 115; 269; 207 | B1; 115; 269; 207 | B1; 115; 269; 207 | 2 |
|  |  | 4 | B1/C12; 115; 269; 203 | B1; 115; 269; 203 | B1; 115; 269; 203 | B1; 115; 269; 203 | 1 |
|  |  | 5 | B1; 115; 269; 207 | B1; 115; 269; 207 | B1; 115; 269; 205 | B1; 115; 269; 207 | 2 |
|  |  | 6 | B1; 115; 269; 207 | B1; 115; 269; 207 | B1; 115; 269; 207 | B1; 115; 269; 207 | 1 |

^a^: Reef abbreviations provided in Fig. 1.

^b^: Data listed as ITS2 “type(s)”, followed by genotype(s) defined as allele sizes for loci CA6.38, B7Sym34, and B7Sym36, respectively. ND (no data) indicates that these alleles from this microsatellite locus failed to amplify successfully after multiple attempts with this sample.

^c^: Sample collected in October 2002 instead of March 2003.

^d^: For locus B7Sym34, the following alleles were detected: 285, 285/293, and 293. Because *Symbiodinium* is a haploid organism [39,40-42], detection of 285/293 in one sample is indicative of a mixed population of two symbiont genotypes rather than a heterozygous genotype. Therefore, this colony was scored as harboring two genotypes rather than three.

^e^: Includes data from the colony sides presented in Table 2.

^f^: Only ITS2 “type” C12 was identified within the detection threshold of DGGE [31]. However, microsatellite analysis confirmed the presence of ITS2 “type” B1 at sub-DGGE detection levels.
